# Unveiling the Silent Threat: The Rise of Β-Lactamase Enzymes in Gram-Negative Bacterial Isolates Identified From Sterile Body Fluids in an Indian Healthcare Institution

**DOI:** 10.7759/cureus.83155

**Published:** 2025-04-28

**Authors:** Rounak Patel, Satyajeet Pawar, Kailash Wagh, Md Abdullah, Prashanth K Guddeti, Bhawani S Verma, Smita S Mundhe, Vaishnavi B Shevale

**Affiliations:** 1 Department of Microbiology, Dr. Ulhas Patil Medical College and Hospital, Jalgaon Khurd, IND; 2 Department of Microbiology, Krishna Vishwa Vidyapeeth (Deemed To Be University), Karad, IND

**Keywords:** ampc beta (β) lactamases, beta-lactamases, extended spectrum beta-lactamase (esbl), gram-negative bacteria (gnb), life-threatening infections, metallo β-lactamases, sterile body fluids

## Abstract

Background

Bacterial infections in sterile body fluids represent a significant clinical concern, particularly when caused by resistant pathogens. β-lactamase-producing gram-negative bacteria, including extended-spectrum-lactamase (ESBL), metallo-β-lactamase (MBL), and AmpC β-lactamase producers, complicate treatment strategies, leading to poor patient outcomes. Infections in vulnerable patients, particularly in intensive care units (ICUs), are more susceptible to these resistant organisms, highlighting the need for urgent surveillance and effective antimicrobial strategies.

Objectives

The primary goal of this study was to assess the prevalence and antimicrobial resistance patterns of bacterial isolates from sterile body fluids, with a focus on β-lactamase-producing gram-negative bacteria. The study further aimed to highlight the implications of antimicrobial resistance patterns in guiding effective empirical therapy and infection control strategies.

Methodology

A total of 180 sterile body fluid samples, including cerebrospinal fluid (CSF), pleural fluid, pericardial fluid, bile, peritoneal or ascitic fluid, and synovial fluid, were collected and processed for bacterial isolation. Standard microbiological procedures, including Gram staining, culture on appropriate media, and biochemical identification tests, were utilized to identify the isolates, followed by antimicrobial susceptibility testing (AST) using the Kirby-Bauer disk diffusion susceptibility test to determine resistance profiles, with particular attention to ESBL, MBL, and AmpC β-lactamase production.

Results

Of the 180 samples, 27 (15%) showed bacterial growth, with *Escherichia coli* and *Pseudomonas aeruginosa* being the most frequently isolated pathogens. Testing for antimicrobial susceptibility showed notable resistance levels to commonly used antibiotics, including cefoperazone-sulbactam and piperacillin-tazobactam. ESBL production was found in 40.74% of the gram-negative isolates, and MBL production was present in 48.15%. The study recorded maximum resistance rates in CSF samples, indicating the critical need for rapid and accurate diagnostic methods. The resistance profiles of isolated pathogens revealed limited options for empirical treatment, underscoring the need for targeted antimicrobial stewardship strategies.

Conclusion

The study underscores the growing concern of multidrug-resistant gram-negative bacteria in sterile body fluid infections, particularly in vulnerable patient populations. The detection of ESBL, MBL, and AmpC-producing organisms highlights the urgency for enhanced surveillance, rapid diagnostics, and strict antimicrobial stewardship to mitigate the impact of these resistant pathogens.

## Introduction

The discovery of β-lactams in the 1940s was a landmark event in the history of medicine, marking the beginning of a new era as part of the therapeutic strategy for bacterial infections. As the second class of antibiotics implemented for therapeutic use, β-lactams significantly advanced healthcare [1]. β-lactam antibiotics are widely used to treat bacterial infections [2]. *Enterobacteriaceae *and oxidase-positive gram-negative bacilli are specifically known for their resistance to β-lactam antibiotics, which is primarily attributed to the production of β-lactamase enzymes, including extended-spectrum β-lactamases (ESBLs), metallo β-lactamases (MBLs), and AmpC β-lactamases, that degrade these drugs [3]. β-lactamases inactivate penicillins, cephalosporins, monobactams, and carbapenems by breaking the amide bond of the β-lactam ring [2].

The demonstration of β-lactamase-producing bacteria in sterile body fluids highlights a substantial threat to the efficacy of β-lactam antibiotics. According to the World Health Organization (WHO), sterility implies the total elimination of all viable microorganisms. In this context, sterile body fluids refer to biological fluids that do not contain any microbial presence, including bacteria that are not part of the normal microbial flora or present as commensals [4,5]. Body fluids such as cerebrospinal fluid (CSF), pleural fluid, pericardial fluid, bile, peritoneal or ascitic fluid, and synovial fluid are generally recognized as sterile under normal physiological conditions, as they are free from viable microorganisms [6]. The presence and multiplication of bacteria in these fluids can lead to severe infections, often associated with high morbidity and mortality rates [7]. In such a case, the prompt detection and identification of pathogens in these fluids, coupled with antimicrobial susceptibility testing, are essential for effective clinical management. Although bacterial growth in these fluids is infrequent due to the lower pathogen load and prior empirical antibiotic treatment, the isolation of even a single colony from such specimens is regarded as indicative of pathogenic microorganisms [8,9].

The growing prevalence of β-lactamase-producing bacteria, especially in sterile body fluids, indicates an urgent challenge in the treatment of infections. These bacteria, which can degrade β-lactam antibiotics, compromise the efficacy of vital drugs used in clinical settings. As such, their presence in sterile fluids, traditionally considered free from microorganisms, signals an alarming trend toward multidrug resistance that demands immediate attention and innovative solutions in both diagnostics and therapeutic strategies. This study aims to explore the prevalence and antimicrobial susceptibility patterns of gram-negative bacterial isolates from sterile body fluids, focusing on β-lactamase-producing bacteria and their implications for clinical treatment protocols. 

## Materials and methods

Location, design, and timeline of the study

This was a cross-sectional, hospital-based study carried out in the Microbiology Department of Krishna Vishwa Vidyapeeth (Deemed To Be University) (Formerly, Krishna Institute of Medical Sciences), Karad, Maharashtra, India, over the duration of one year, from November 2022 to November 2023.

Sample size estimation and focus on β-lactamase producers

The study’s primary objective was to identify bacterial isolates derived from sterile body fluids and evaluate their potential for β-lactamase production, a significant mechanism of antimicrobial resistance. To estimate the required sample size, the formula $n=\frac{4pq}{l^{2}}$ was applied, where p denotes the prevalence of bacterial growth, q is 100-p, and I is the desired precision. Based on the study conducted by Sharma et al. [10], the prevalence rate was found to be 30%.

$n=\frac{4pq}{l^{2}}$

= $\frac{4 &times; 30 &times; 70}{72}$

= 171

Thus, a minimum of 171 sterile body fluid specimens was required, but 180 samples were included to enhance the study’s robustness. Specimens were collected consecutively, ensuring unbiased representation. The study’s design emphasized detecting β-lactamase enzyme producers, critical in guiding effective therapeutic strategies and combating rising antimicrobial resistance.

Ethical clearance

Ethical approval for the study was obtained from the Institutional Ethics Committee (IEC) of Krishna Institute of Medical Sciences. The approval was issued under protocol number 069/2021-2022 and reference number KIMSDU/IEC/04/2022, dated May 9, 2022.

Inclusion and exclusion criteria

After approval by the IEC, sterile body fluid specimens, excluding blood and urine, collected from hospitalized patients at Krishna Hospital, Karad, were included in the study. Specimens were selected irrespective of the patient's age or gender. Samples were excluded if transported more than two hours after collection or if the patient had received antibiotic treatment within the preceding two weeks.

Procedures for specimen collection and processing

Sterile body fluid specimens comprising CSF, pleural fluid, pericardial fluid, bile, peritoneal or ascitic fluid, and synovial fluid were collected from patients following informed consent. All samples were collected in sterile containers and promptly transported to the microbiology laboratory within two hours of being collected to maintain sample integrity.

In the laboratory, standard microbiological procedures were applied for the analysis of the specimens. Smear preparation was performed directly from the specimen for Gram staining. Subsequently, the specimens were cultured on both enriched media, such as blood agar and heated blood agar, and selective/differential media like MacConkey agar (HiMedia Laboratories Private Limited, Mumbai, India) using the four-quadrant streaking technique. The inoculated plates were incubated at 37°C for 24 hours, followed by observation for bacterial growth after the incubation period. The growth was characterized based on colony morphology, Gram stain reaction, motility, and biochemical profiles for species identification. Samples showing no growth after 48 hours of incubation were classified as sterile [11]. 

In cases where isolates belonged to the genus *Pseudomonas*, *P. aeruginosa* was identified based on characteristic pigment production, growth at 42°C, and standard biochemical reactions. Isolates not fulfilling these criteria but still consistent with the genus were reported as *Pseudomonas *species (non-aeruginosa), as species-level differentiation beyond *P. aeruginosa* was not feasible with conventional methods alone.

Antimicrobial susceptibility testing

Antimicrobial susceptibility testing for each isolate was performed utilizing the disc diffusion technique as per the Kirby-Bauer method, complying with the guidelines established by the Clinical and Laboratory Standards Institute (CLSI), 2023 [12]. The antibiotics used for testing included amikacin (30 μg), gentamicin (10 μg), ciprofloxacin (5 μg), cefotaxime (30 μg), imipenem (10 μg), cefoxitin (30 μg), ceftazidime (30 μg), piperacillin-tazobactam (75/30 μg), trimethoprim/sulfamethoxazole (1.225/23.75 μg), and fosfomycin (200 μg). Zone diameters were interpreted according to the CLSI 2023 interpretive criteria. *Escherichia coli* ATCC 25922 was used as a quality control strain to ensure the accuracy and reproducibility of the test results.

Phenotypic detection of β-lactamase enzymes in gram-negative bacterial isolates

Phenotypic tests were used to identify the presence of various β-lactamase enzymes, which play a crucial role in the resistance of gram-negative bacteria to β-lactam antibiotics. Specifically, the detection focused on ESBLs, MBLs, and AmpC β-lactmases. These enzymes impart resistance to critical antibiotics, complicating the treatment of infections and posing a significant challenge in clinical settings.

Detection of ESBLs

ESBLs are enzymes that hydrolyze a wide range of β-lactam antibiotics, including third-generation cephalosporins. To detect ESBL-producing strains, ceftazidime and a combination of ceftazidime with clavulanic acid (a β-lactamase inhibitor) were used. Due to resource limitations, cefotaxime and cefotaxime-clavulanate combination discs could not be included; however, ceftazidime and ceftazidime-clavulanate remain acceptable for ESBL screening as per CLSI M100 guidelines when used with appropriate interpretative criteria. The principle behind this test is that clavulanic acid inhibits the activity of ESBLs, resulting in an increased zone of inhibition around the ceftazidime-clavulanic acid combination disc. A significant difference in zone size of >5 mm between the ceftazidime and the ceftazidime-clavulanic acid discs is considered indicative of ESBL production. This method is commonly used in clinical microbiology laboratories as a standard approach to identify resistance patterns [12,13].

Detection of MBLs

MBLs are a class of β-lactamases that require zinc ions for their enzymatic activity, enabling them to hydrolyze a broad range of β-lactam antibiotics, including carbapenems. MBL identification was accomplished through the imipenem combined with ethylenediaminetetraacetic acid (EDTA), which chelates the zinc ions and inhibits MBL activity. A significant difference of >7 mm in the zone of inhibition between imipenem and imipenem-EDTA discs was used to confirm MBL production. This method is based on the ability of EDTA to specifically inhibit MBLs, thus helping distinguish between carbapenem-resistant isolates due to β-lactamase production or other mechanisms [12,14].

AmpC β-lactamase detection

AmpC β-lactamases are enzymes that can hydrolyze a wide variety of β-lactam antibiotics, except carbapenems. These enzymes are often plasmid-mediated and can lead to resistance against penicillins, cephalosporins, and cephamycins. The demonstration of AmpC β-lactamase was carried out using the cefoxitin disc, both alone and in combination with cloxacillin, which is a β-lactamase inhibitor. A >4 mm difference in the inhibition zone between the cefoxitin and cefoxitin-cloxacillin disks is considered significant and indicative of AmpC β-lactamase production. The use of cloxacillin helps in distinguishing AmpC producers from non-producers, as it inhibits β-lactamase activity [15,16].

Statistical analysis

The data obtained in this study were entered and organized using Microsoft Excel (Microsoft Corporation, Redmond, Washington, United States). Descriptive statistics, including numbers (n) and percentages (%), were used to summarize the findings and were presented in the form of tables and graphs. The IBM SPSS Statistics for Windows, Version 28.0 (Released 2021; IBM Corp., Armonk, New York), was used to perform the chi-square test for evaluating associations, and a p-value <0.05 was considered statistically significant.

## Results

A total of 180 samples of sterile body fluid were collected for culture and sensitivity testing. Among the patients, 74.45% were male and 25.55% were female. The distribution of sample types included CSF (48 samples, 26.67%), pleural fluid (53 samples, 29.45%), peritoneal (ascitic) fluid (73 samples, 40.56%), pericardial fluid (four samples, 2.22%), and one sample each of bile and synovial fluid (0.55% of the total), which are too few in number to allow meaningful interpretation.

Of the total specimens, bacterial growth was observed in 27 cases (15%). Peritoneal (ascitic) fluid exhibited the highest bacterial growth rate (n=15, 20.55%), while no growth was detected in the bile and synovial fluid samples (Table 1). 

**Table 1:** Distribution of bacterial growth across various sterile body fluids CSF: cerebrospinal fluid

Body Fluids	Total number (Percentage)	Growth, n (%)	No growth, n (%)
CSF	48 (26.67)	2 (4.17)	46 (95.83)
Pleural Fluid	53 (29.45)	9 (16.99)	44 (83.01)
Peritoneal (Ascitic) Fluid	73 (40.56)	15 (20.55)	58 (79.45)
Pericardial Fluid	4 (2.22)	1 (25)	3 (75)
Bile	1 (0.55)	0 (0)	1 (100)
Synovial Fluid	1 (0.55)	0 (0)	1 (100)
Total	180	27 (15)	153 (85)

**Figure 1:**
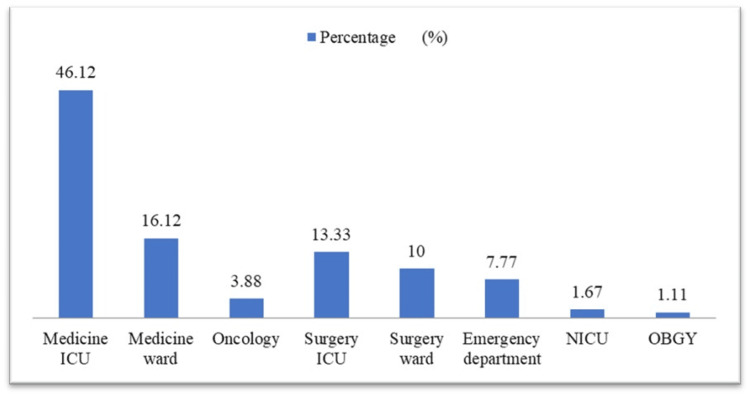
Distribution of body fluid specimens among hospital wards (N=180) ICU: intensive care unit; NICU: neonatal intensive care unit; OBGY: obstetrics and gynecology

The highest number of sterile body fluid samples was collected from the medicine ICU, comprising 83 samples (46.12%), followed by 29 samples (16.12%) from the medicine ward. Contributions from the surgery ICU accounted for 24 samples (13.33%), while the surgery ward and emergency department provided 18 (10%) and 14 (7.77%) samples, respectively. Other departments, including oncology (seven samples, 3.88%), neonatal intensive care unit (NICU) (three samples, 1.67%), and obstetrics and gynaecology (two samples, 1.11%), accounted for smaller proportions, as depicted in Figure 1. 

Of the 27 culture-positive samples, *E. coli *was the predominant organism isolated (n=9, 33.33%), followed by *P. aeruginosa* (n=6, 22.22%), *Klebsiella pneumoniae* (n=4, 14.81%), *Acinetobacter baumannii *(n=3, 11.12%), *Enterobacter cloacae* (n=2, 7.41%) and non-aeruginosa *Pseudomonas *species(n=3, 11.12%) that could not be identified to the species level due to limited resources for advanced identification methods (Table 2).

**Table 2:** Bacterial isolates identified in different sterile body fluids CSF: cerebrospinal fluid

Bacterial Isolates	CSF, n (%)	Pleural fluid, n (%)	Peritoneal Fluid, n (%)	Pericardial Fluid, n (%)	Total, n (%)
Escherichia coli	-	4 (44.44)	5 (55.56)	-	9 (33.33)
Pseudomonas aeruginosa	2 (33.34)	1(16.66)	2 (33.34)	1 (16.66)	6 (22.22)
Klebsiella pneumoniae	-	-	4 (100)	-	4 (14.81)
Acinetobacter baumannii	-	2 (75)	1 (25)	-	3 (11.12)
Enterobacter cloacae	-	-	2 (100)	-	2 (7. 40)
Pseudomonas species	-	2 (75)	1 (25)	-	3 (11.12)
Total	2 (7.40)	9 (33.34)	15 (55.56)	1 (3.70)	27

Among the bacterial isolates derived from sterile body fluids,* E. coli* exhibited the highest prevalence. It demonstrated maximum susceptibility to gentamicin and fosfomycin (77.77%) but showed a high resistance to cefoperazone-sulbactam (88.88%). *P. aeruginosa* exhibited high susceptibility towards fosfomycin (83.34%) and displayed significant resistance to gentamicin, cefotaxime, and cefoxitin (83.34%). Notably, in line with CLSI guidelines (2023), co-trimoxazole is not recommended for susceptibility testing against *Pseudomonas* species due to their intrinsic resistance to the drug and has therefore been excluded from interpretation. *K. pneumoniae* exhibited 50% susceptibility to amikacin, cefoxitin, and ceftazidime, with complete resistance to cefotaxime. *A. baumanni* showed 100% sensitivity to co-trimoxazole and fosfomycin but was fully resistant to gentamicin and cefotaxime. *Pseudomonas* species had maximum sensitivity to amikacin, ceftazidime, and fosfomycin (66.66%) and resistance to gentamicin, ciprofloxacin, and cefotaxime (66.66%). *E. cloacae* was highly sensitive to co-trimoxazole and fosfomycin (100%) but exhibited full resistance to imipenem, cefoxitin, and ceftazidime as presented in Table 3. 

**Table 3:** Antimicrobial susceptibility pattern of bacterial isolates S: sensitive; R: resistant; NT: not tested

Antibiotics	*Escherichia coli*		*Pseudomonas aeruginosa *		Klebsiella pneumoniae		Acinetobacter baumannii		*Pseudomonas *species		Enterobacter cloacae	
	S	R	S	R	S	R	S	R	S	R	S	R
Amikacin	44.44%	55.56%	66.67%	33.33%	50%	50%	33.34%	66.66%	66.66%	33.34%	50%	50%
Gentamicin	77.77%	22.23%	16.66%	83.34%	25%	75%	0%	100%	33.34%	66.66%	50%	50%
Ciprofloxacin	33.34%	66.66%	33.33%	66.67%	25%	75%	33.34%	66.66%	33.34%	66.66%	50%	50%
Cefotaxime	22.23%	77.77%	16.66%	83.34%	0%	100%	0%	100%	33.34%	66.66%	50%	50%
Imipenem	22.23%	77.77%	33.33%	66.67%	25%	75%	66.66%	33.34%	33.34	(66.66)	0%	100%
Cefoxitin	33.34%	66.66%	16.66%	83.34%	50%	50%	66.66%	33.34%	0%	100%	0%	100%
Ceftazidime	0%	100%	16.66%	83.34%	50%	50%	33.34%	66.66%	66.66%	33.34%	0%	100%
Piperacillin-tazobactam	22.23%	77.77%	16.66%	83.34%	25%	75%	33.34%	66.66%	66.66%	33.34%	50%	50%
Cefoperazone- sulbactam	11.12%	88.88%	33.33%	66.67%	50%	50%	33.34%	66.66%	33.34%	66.66%	50%	50%
Co-trimoxazole	33.34%	66.66%	NT	NT	50%	50%	100%	0%	NT	NT	100%	0%
Fosfomycin	77.77%	22.23%	83.34%	16.66%	50%	50%	100%	0%	66.66%	33.34%	100%	0%

Of the 27 bacterial isolates, *E. coli* (n=6, 66.67%) emerged as the predominant MBL producers, followed by *K. pneumoniae* (n=2, 50%), *P. aeruginosa* (n=2, 33.33%), and *Pseudomonas* species (n=1, 33%). Notably, *E. cloacae* did not exhibit MBL production. Overall, 13 isolates (48.15%) were identified as MBL producers (Table 4). 

**Table 4:** Incidence of metallo-beta-lactamase (MBL) producing bacterial pathogens MBL: metallo-beta lactamase

Bacterial isolates	MBL Positive, n (%)	MBL Negative, n (%)
Escherichia coli	6 (66.67)	3 (33.33)
Pseudomonas aeruginosa	2 (33.33)	4 (66.67)
Klebsiella pneumoniae	2 (50)	2 (50)
Acinetobacter baumannii	2 (67)	1 (33)
Enterobacter cloacae	0 (0)	2 (100)
*Pseudomonas* species	1 (33)	2 (67)

Out of the 27 bacterial isolates, *E. coli* (n=5, 55.56%) was the most prevalent ESBL producer, followed by* E. cloacae* (n=2,100%), *P. aeruginosa *(n=2, 33.33%), *K.* *pneumoniae *(n=1, 25%), and* Pseudomonas* species (n=1, 33%). *Acinetobacter baumannii*, however, did not exhibit ESBL production. In total, 11 isolates (40.74%) were identified as ESBL producers (Table 5). 

**Table 5:** Occurrence of extended-spectrum-beta-lactamase (ESBL) producing bacterial isolates ESBL: extended-spectrum-beta-lactamase

Bacterial isolates	ESBL Positive, n (%)	ESBL Negative, n (%)
Escherichia Coli	5 (55.56)	4 (44.44)
Pseudomonas aeruginosa	2 (33.33)	4 (66.67)
Klebsiella pneumoniae	1 (25)	3 (75)
Acinetobacter baumannii	0 (0)	3 (100)
Enterobacter cloacae	2 (100)	0 (0)
*Pseudomonas* species	1 (33)	2 (67)

Of the 27 bacterial isolates, *P. aeruginosa *(n=2, 33.33%), *K. pneumoniae* (n=2, 50%), and *Pseudomonas* species (n=1, 33%) were identified as AmpC β-Lactamase producers. In contrast, *E. coli*, *A. baumannii*, and *E. cloacae* were non-AmpC producers. The total prevalence of AmpC producers in this study was five isolates (18.51%) (Table 6). 

**Table 6:** Frequency of AmpC beta-lactamase producing isolates AmpC: ampicillin cephalosporinase

Bacterial isolates	AmpC Positive, n (%)	AmpC Negative, n (%)
*Escherichia *Coli	0 (0)	9 (100)
Pseudomonas aeruginosa	2 (33.33)	4 (66.67)
Klebsiella pneumoniae	2 (50)	2 (50)
Acinetobacter baumannii	0 (0)	3 (100)
Enterobacter cloacae	0 (0)	2 (100)
*Pseudomonas* species	1 (33)	2 (67)

**Figure 2:**
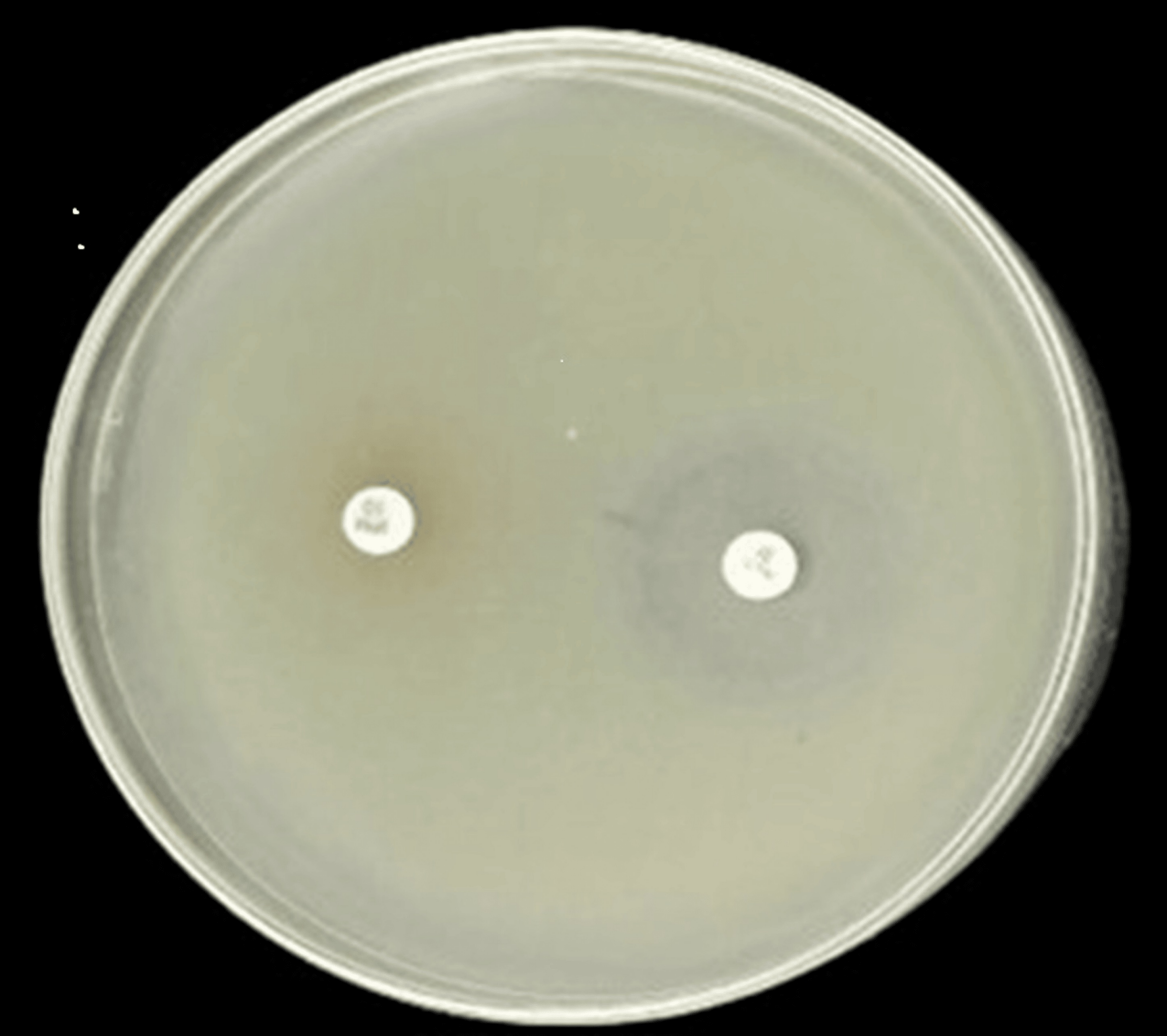
Phenotypic detection of MBL-producing gram-negative bacterial isolate MBL: metallo-beta lactamase

Phenotypic identification of MBL-producing gram-negative bacteria using the imipenem-EDTA combined disk diffusion assay is illustrated in Figure 2. 

**Figure 3:**
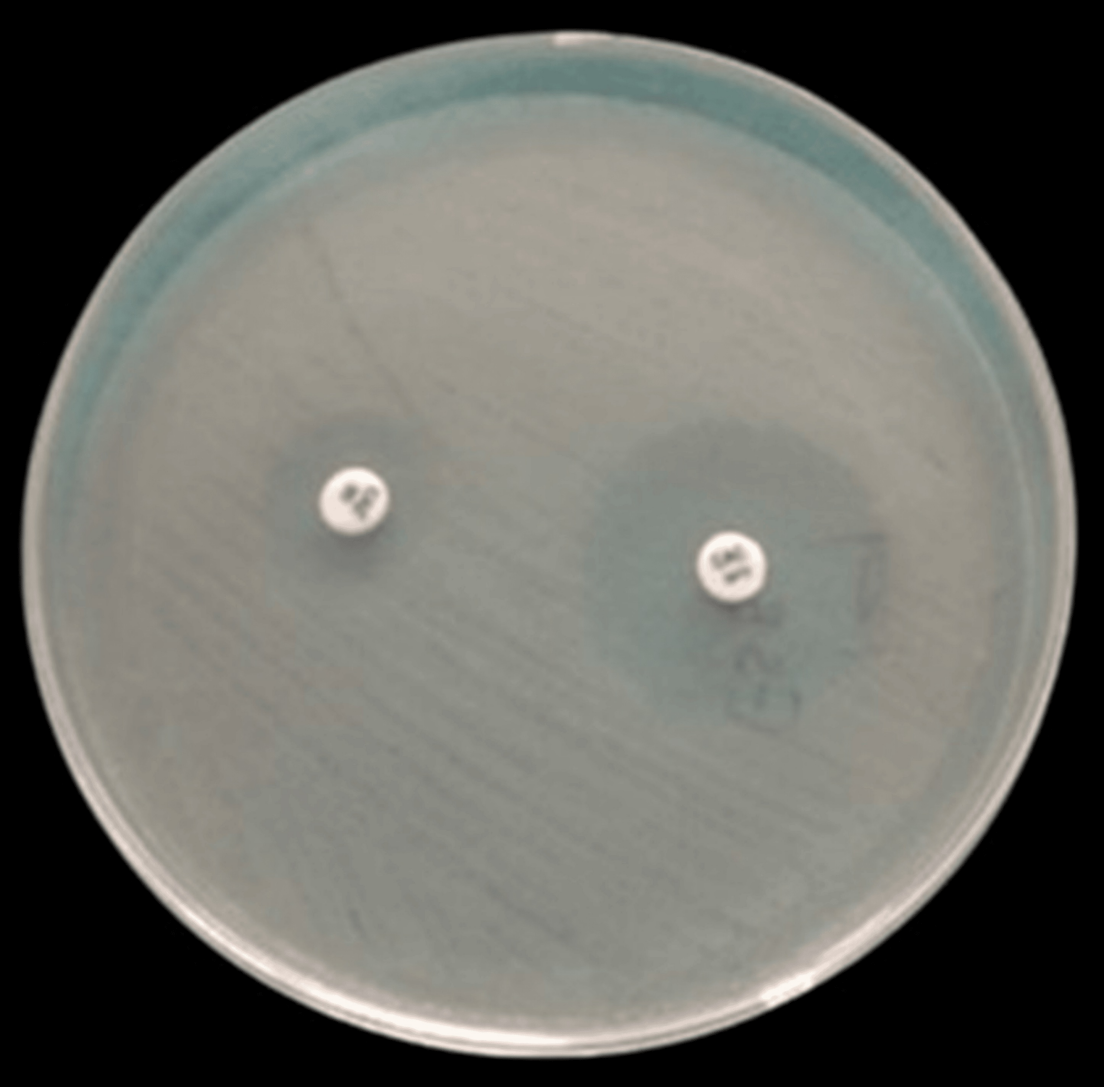
Phenotypic detection of ESBL producing gram-negative bacterial isolate ESBL: extended-spectrum-beta lactamase

Figure 3 depicts the phenotypic detection of ESBL-producing gram-negative bacteria using ceftazidime + clavulanic acid as a combined disk diffusion method. 

**Figure 4:**
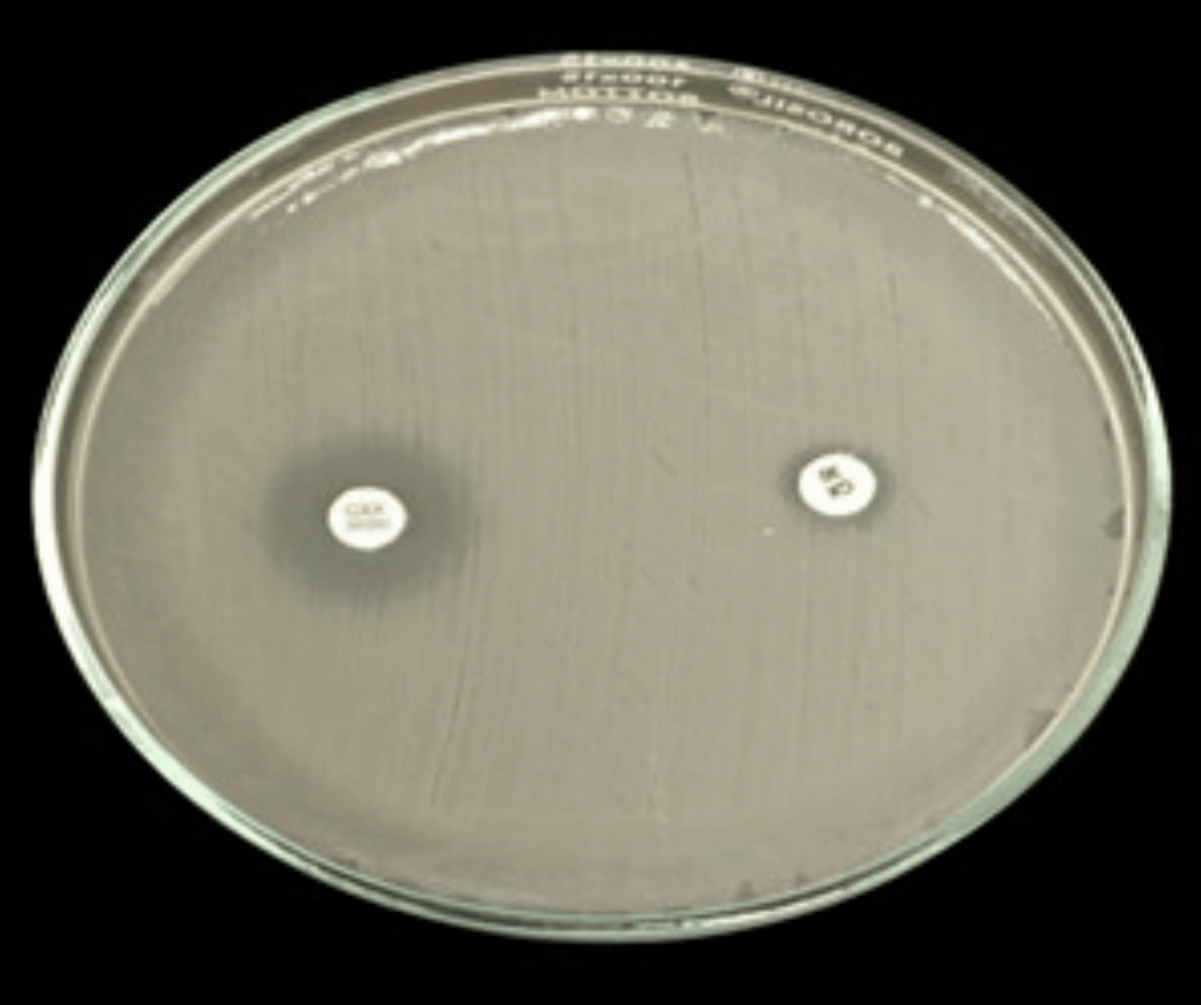
Phenotypic detection of AmpC beta lactamase producing gram-negative bacterial isolate AmpC: ampicillin cephalosporinase

Phenotypic detection of AmpC β-lactamase-producing gram-negative bacteria using the cefoxitin-cloxacillin combined disk diffusion method is depicted in Figure 4. 

## Discussion

Systemic illnesses can occur when microorganisms invade sterile body sites, disrupting the normal physiology and potentially leading to severe clinical outcomes [17]. The detection of microorganisms, including bacteria, viruses, fungi, and parasites (both protozoa and metazoa), from these sterile sites is a concerning finding, as their presence is often harmful and may escalate into life-threatening conditions. This is particularly critical in vulnerable patients, such as those admitted to ICUs or high-dependency units (HDUs), where compromised immunity and critical illness increase susceptibility to infection [18]. Building upon these concerns, this study sheds light on the growing threat of β-lactamase-producing gram-negative bacteria isolated from sterile body fluids. The presence of enzymes like ESBL, MBL, and AmpC significantly complicates infection management due to their role in antibiotic resistance. Our findings emphasize the urgent need for robust surveillance, rapid diagnostic techniques, and effective antimicrobial stewardship strategies to mitigate these emerging resistance patterns and improve clinical outcomes for affected patients.

As per the current study, the overall prevalence rate of bacterial etiological agents was observed to be 15%, which is comparable to several previous studies. For instance, a study by Durga et al. in Telangana, India, reported a prevalence rate of 20.55%, which is slightly higher than our findings [19]. Studies from Ethiopia reported similar prevalence rates; Shume et al. [17] in Eastern Ethiopia reported 17%, while Tsegay et al. [20] in Northern Ethiopia reported 20.2%. On the other hand, studies with lower prevalence rates have been reported, for example by Admas et al. in in Northwest Ethiopia (7.5%) [21], by Duran et al. in in Balikesir, Turkey (9.7%) [6], and by Singh et al. in Uttar Pradesh, India (9.69%) [22]. Contrastingly, higher prevalence rates have been documented by Kar et al. in Northern India (31.13%) [23], Shrestha et al. in Sunsari, Nepal (31%) [8], and Tiwari et al. in Odisha, India (28%) [18]. These variations in prevalence rates are likely due to procedural differences in how samples were processed, seasonal factors, and regional variations in infection control practices across various studies.

Of the 32 culture-positive samples in the current study, *E. coli* was the most commonly isolated organism, accounting for 33.33%, followed by *P. aeruginosa* at 22.22%. Similar findings were reported by other studies. For instance, Rouf et al. [5], Shrestha et al. [8], and Durga et al. [19] identified *E. coli* as the predominant pathogen in sterile body fluids, reflecting consistent trends in its isolation rate across different settings. These observations highlight the critical role of *E. coli* as the leading cause of infections in sterile body fluids, emphasizing the necessity of targeted interventions and appropriate antimicrobial management strategies.

The isolated organisms were examined against various antimicrobial agents to determine their susceptibility patterns. *E. coli*, the dominant isolate, demonstrated the highest susceptibility to gentamicin and fosfomycin at 77.78%, followed by amikacin at 44.44%. The results are in line with the observations made by Durga et al., who reported 85% susceptibility to amikacin and 75% to gentamicin [19]. Similarly, a study by Rouf et al. highlighted that gentamicin and amikacin were among the most potent antibiotics against *E. coli* [5].

Conversely, *E. coli *showed the highest resistance to cefoperazone-sulbactam at 88.88%, followed by cefotaxime, imipenem, and piperacillin-tazobactam at 77.77%. This pattern is consistent with the findings of Sheikhbahaei et al., who observed significant resistance to piperacillin-tazobactam and cefoperazone-sulbactam [24].

*P. aeruginosa*, the second most common isolate, exhibited maximum sensitivity to fosfomycin at 83.34%, followed by amikacin at 66.67%. These results mirror those mentioned by Shume et al. [17] and Durga et al. [19], where amikacin sensitivity was noted at 75%.

As per the present study, ESBL- and MBL-producing gram-negative bacteria were notably detected in CSF samples, highlighting a critical concern for antimicrobial resistance in life-threatening infections. Overall, 40.74% of the bacterial isolates were ESBL producers, similar to findings reported by Shrestha et al. (37%) [8] and Singh et al. (25%) [22]. Additionally, MBL production was observed in 48.15% of cases, with CSF samples contributing significantly to these resistant strains. Among the 27 gram-negative isolates, 18.51% exhibited AmpC β-lactamase production.

The detection of ESBL- and MBL-producing bacteria in CSF samples underlines the severity of resistance mechanisms in central nervous system infections, where timely and effective antimicrobial therapy is critical. The ability of ESBL- and MBL-producing bacteria to proliferate in sterile body fluids like CSF can be attributed to multiple factors. These include the increasing number of immunocompromised patients, such as those with malignancy, undergoing neurosurgery, or with indwelling devices, who are more susceptible to infections [25]. The potent resistance mechanisms of these bacteria, such as production of β-lactamases and carbapenemases, enable them to survive and multiply even in the presence of commonly used antibiotics [13]. Furthermore, biofilm formation on medical devices can protect bacteria from both the host immune response and antimicrobial agents, facilitating persistence in sterile compartments. Invasive procedures and breaches in the blood-brain barrier can also serve as entry points for these organisms into the CSF. Delayed diagnosis and inadequate empirical therapy further contribute to their unchecked proliferation and worsened outcomes [26].

The rising prevalence of AmpC β-lactamase, MBL, and ESBL-producing isolates reflects a worrying trend of escalating resistance mechanisms in bacteria, which poses a serious challenge to the effectiveness of current antimicrobial therapies [27]. The high proportion of multidrug-resistant organisms, particularly ESBL and MBL producers in CSF samples, underscores the need for early diagnosis and empirical treatment adjustments in critically ill patients. These findings reinforce the clinical importance of integrating microbiological data into patient management strategies, especially in ICU and HDU settings.

Limitations

This study had certain limitations that should be acknowledged. Firstly, only conventional culture and phenotypic methods were employed, which might have limited the detection of fastidious organisms, anaerobes, and certain resistant strains not easily identified without molecular techniques. Secondly, molecular characterization of β-lactamase genes was not performed, which could have provided more precise insights into resistance mechanisms. Additionally, the study was conducted at a single tertiary care center, which may limit the generalizability of the findings to other geographical regions or healthcare settings. Finally, the study did not correlate clinical outcomes with microbiological findings, which could have further strengthened the clinical relevance of the results. Moreover, the extremely limited number of bile and synovial fluid samples (only one each) precludes any meaningful interpretation or generalization of findings related to these specimen types.

Future recommendations

To enhance the clinical relevance of bacteriological findings, future research should aim to incorporate detailed clinical parameters such as patient comorbidities, prior antibiotic exposure, ICU admission status, and treatment outcomes. This integrated approach would provide a more comprehensive understanding of the risk factors associated with infections caused by β-lactamase-producing gram-negative bacteria in sterile body fluids, ultimately supporting more targeted therapeutic and infection control strategies.

## Conclusions

This study highlights the growing challenge of multidrug-resistant gram-negative bacteria in sterile body fluids, especially *E. coli* and *P. aeruginosa*. The detection of ESBL, MBL, and AmpC producers emphasizes the need for timely microbiological diagnosis to guide appropriate therapy. Strengthening antimicrobial stewardship programs and updating empirical treatment guidelines based on local susceptibility trends are crucial. Moreover, robust infection control measures should be reinforced to curb the spread of resistant pathogens. Future research involving molecular surveillance can provide deeper insights into resistance mechanisms and enhance early detection efforts.
